# Oral administration of curcumin and quercetin nanoparticles can improve ulcerative colitis by regulating intestinal microorganisms

**DOI:** 10.3389/fnut.2025.1696699

**Published:** 2025-10-01

**Authors:** Yingxi Li, Zhiyue Xu, Shiyang Zhao, Tianyi Huang, Jianzhong Xu, Sitong Wang, Yaxin Sang, Wenlong Yu, Xianghong Wang

**Affiliations:** ^1^College of Food Science and Technology, Hebei Agricultural University, Baoding, China; ^2^Chenguang Biotech Group Co., Ltd., Handan, China

**Keywords:** zein, curcumin, quercetin, co-encapsulation, bioavailability, ulcerative colitis

## Abstract

**Background:**

Curcumin has been proved to relieve ulcerative colitis (UC). However, the premature release of gastrointestinal tract will lead to insufficient accumulation of curcumin in colon and affect the improvement effect. Therefore, we developed a kind of oral nanoparticles (Zein-CS-Cur-Que), which made quercetin promote the absorption of curcumin in colon by efflux carrier and inhibiting metabolic enzymes, and at the same time encapsulated curcumin and quercetin with zein and sodium caseinate to promote the targeted release in colon.

**Methods:**

3.5% dextran sodium sulfate was used to establish ulcerative colitis (UC) model in mice, and the improvement effect of nanoparticles on ulcerative colitis was evaluated by comprehensive basic score (weight change rate, DAI score, liver index, and spleen index), inflammatory factors (TNF-α, IL-1β, IL-6, IL-10, and IFN-γ) and oxidative stress (MDA, SOD, CAT, and MPO). The intestinal flora was sequenced by 16S rRNA, and the secretion of short-chain fatty acids was determined by GC-MS. Immunohistochemistry (ZO-1, Claudin-1, and Occludin) and real-time quantitative reverse transcription PCR (qRT-PCR) (TLR4/NF-κB and JAK2/STAT3) were used to analyze the expression of related genes.

**Results:**

The results showed that Zein-CS-Cur-Que can reduce pro-inflammatory factors (INF-γ, IL-6, IL-1β, and TNF-α), increase anti-inflammatory factor (IL-10), up-regulate the expression level of tight junction proteins (ZO-1, Claudin-1, and Occludin) to restore the intestinal barrier, and down-regulate the expression of related protein genes in TLR4/NF-κB and JAK2/STAT3. Ultimately, modulating the relative abundance of Firmicutes and Bacteroidetes promotes the production of short-chain fatty acids and reverses the damage caused by ulcerative colitis.

**Conclusion:**

Nanoparticles (Zein-CS-Cur-Que) can significantly reduce inflammatory reaction, restore intestinal barrier, regulate the expression of related protein genes in TLR4/NF-κB and JAK2/STAT3 pathways, and significantly improve ulcerative colitis. Furthermore, the construction of Zein-CS-Cur-Que provides a scientific basis for increasing curcumin targeting and ameliorating ulcerative colitis.

## 1 Introduction

Ulcerative colitis (UC) is one of the primary inflammatory bowel diseases with clinical manifestations of bloody diarrhea and abdominal pain (1, 2). In clinical practice, treatment usually consists of administration using amino salicylates, glucocorticoids and immunosuppressive agents (3, 4). However, the treatment often suffers from challenges such as high side effects, lack of selectivity, poor specificity, and difficulty in reaching the colon when the drug is released too quickly (5, 6). Oral delivery system relies on the complementary functions of different nanocarriers and drug synergism, which can break through the double barrier of gastric acid and intestinal enzymes, and realize multi-layer targeted delivery, slow release and targeted release of drugs, improve drug bioavailability and multi-level drug distribution, and reduce the drug toxicity and side effects (7, 8). The oral drug delivery system based on nanotechnology has unique advantages and good application prospects in anti-inflammatory and anti-tumor delivery (9, 10).

Zein, a natural polymer found mainly in corn endosperm, can be structurally transformed from α-helix to β-folding by solvent-resistant methods, then assembled separately or with other biopolymers to form nanomaterials such as films, particles, nanoparticles, nanofibers and microcapsules material, which facilitates the encapsulation and release of hydrophobic substances (11, 12). Sodium caseinate, a major protein found in milk, can be used to modify and stabilize zein due to its electrostatic and spatial stabilization properties, resulting in zein-sodium caseinate nanoparticles with high stability and loading capacity (13, 14). This combination has been shown to be effective in delivering bioactive ingredients and enhancing the water solubility and storage stability of drugs (15, 16).

**Scheme 1:**
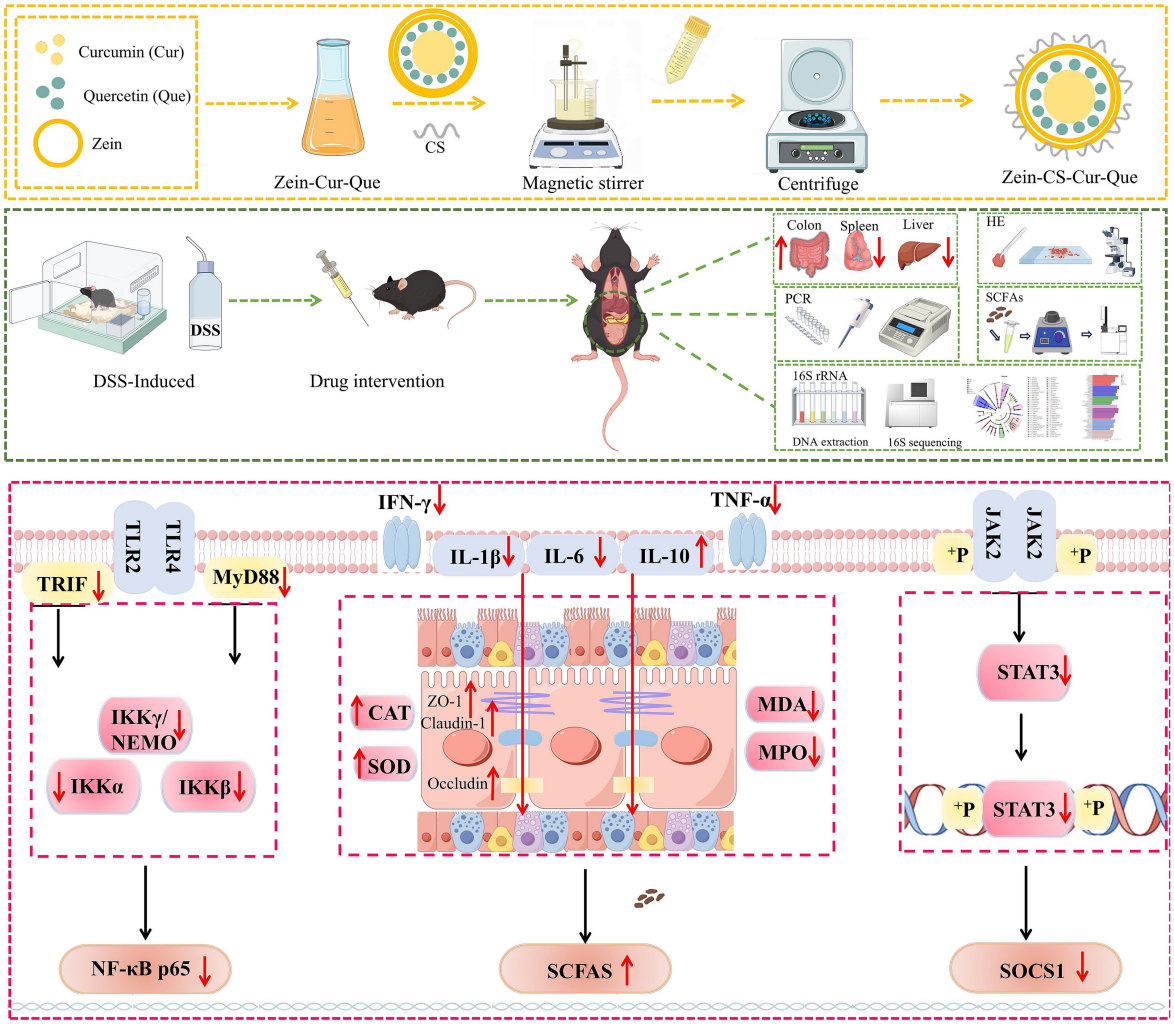
Preparation process of Zein-CS-Cur-Que and schematic diagram of mechanism of treating UC.

Curcumin, a plant polyphenolic compound, is an orange-yellow crystalline powder extracted from the tuberous roots and rhizomes of turmeric (17). Studies at home and abroad show that curcumin can alleviate ulcerative colitis by regulating the expression of cytokines, inhibiting the activation of NF-κB, regulating the polarization of M1/M2 cells, and balancing the activation of TLR signaling pathway, which has great application potential in the treatment of ulcerative colitis. However, the low solubility and high metabolism of curcumin in the body affects digestion, absorption and metabolism in the gastrointestinal tract, thus the research and development of oral delivery systems is of particular importance (18, 19). Quercetin is a flavonol, mainly found in vegetables such as onions and apples, which is readily soluble in organic reagents and possesses antioxidant activity, anti-inflammatory and anti-tumor effects (20, 21). Most importantly, quercetin can inhibit metabolic enzymes and efflux transporters, thus realizing multi-target regulation, metabolic complementation and intestinal flora interactions, and improving the anti-inflammatory activity of curcumin (22, 23). Therefore, in the present study, curcumin and quercetin synergistically delivered nanoparticles were prepared using zein and sodium caseinate as wall materials, and explored the therapeutic mechanism of nanoparticles for UC by establishing a UC model in mice (Scheme 1).

## 2 Materials and methods

### 2.1 Materials

Curcumin (>98%) and quercetin (>98%) were produced from Chenguang Biotechnology Group (Handan, China), while zein and sodium caseinate were sourced from Macklin (Shanghai, China). 5-amino-salicylic acid and the fecal occult blood kit were acquired from Solepol Technology Co., Ltd. (Beijing, China). Dextran sulfate sodium (DSS) was obtained from RuiDa HengHui Science (Beijing, China) and curcumin capsules were purchased from Supherb (Nazaret, Israel). Tumor necrosis factor-α (TNF-α), interferon-γ (IFN-γ), myeloperoxidase (MPO), interleukin-1β (IL-1β), interleukin-6 (IL-6) and interleukin-10 (IL-10) were obtained from Mlbio (Shanghai, China). Superoxide dismutase (SOD), catalase (CAT), malondialdehyde (MDA) and bicinchoninic acid (BCA) kits were purchased from Nanjing Jiancheng Bioengineering (Nanjing, China), Zonula occludens-1 (ZO-1), Occludin-1 and Claudin antibodies were sourced from Proteintech (Wuhan, China). The animal tissue RNA extraction kit, reverse transcription kit, and SYBR Green kit were procured from Tian Gen Biochemical Technology (Beijing, China).

### 2.2 Preparation of nanoparticles

Using zein as carrier and sodium caseinate as stabilizer, nanoparticles carrying curcumin, quercetin were prepared by anti-solvent method. (I) A total of 50 mg of zein and 25 mg of curcumin were mixed and dissolved in 10 mL of 70% ethanol. (II) 50 mg of zein, 25 mg of curcumin, and 5 mg of quercetin were combined and dissolved in 10 mL of 70% ethanol. Subsequently, this mixture (I/II) was, respectively, added into 30 mL of ultrapure water containing 100 mg of sodium caseinate using a syringe, under conditions of rapid magnetic stirring. Following continuous stirring for 2 h, the ethanol in the nanoparticle suspension was removed using a rotary evaporator (24). Concentrate in a volumetric flask to about 25 mL. The final mass ratio of diverse materials within Nanoparticles was CS:Zein:Cur = 20:10:5 and CS:Zein:Cur:Que = 20:10:5:1. The former product was named Zein-CS-Cur, while the latter component was named Zein-CS-Cur-Que, which was consistent with the names of subsequent animal experiments.

### 2.3 DSS-induced ulcerative colitis mouse model and dietary intervention

Male C57BL/6 mice (6 weeks old; weight: 20 ± 2 g) were purchased from Beijing SPF Biotechnology Co., Ltd. All animal experiments in this study followed the guidelines of the Hebei Agricultural University (HABU) and approved by the Animal Ethics Committee (2023197).

**Figure 1:**
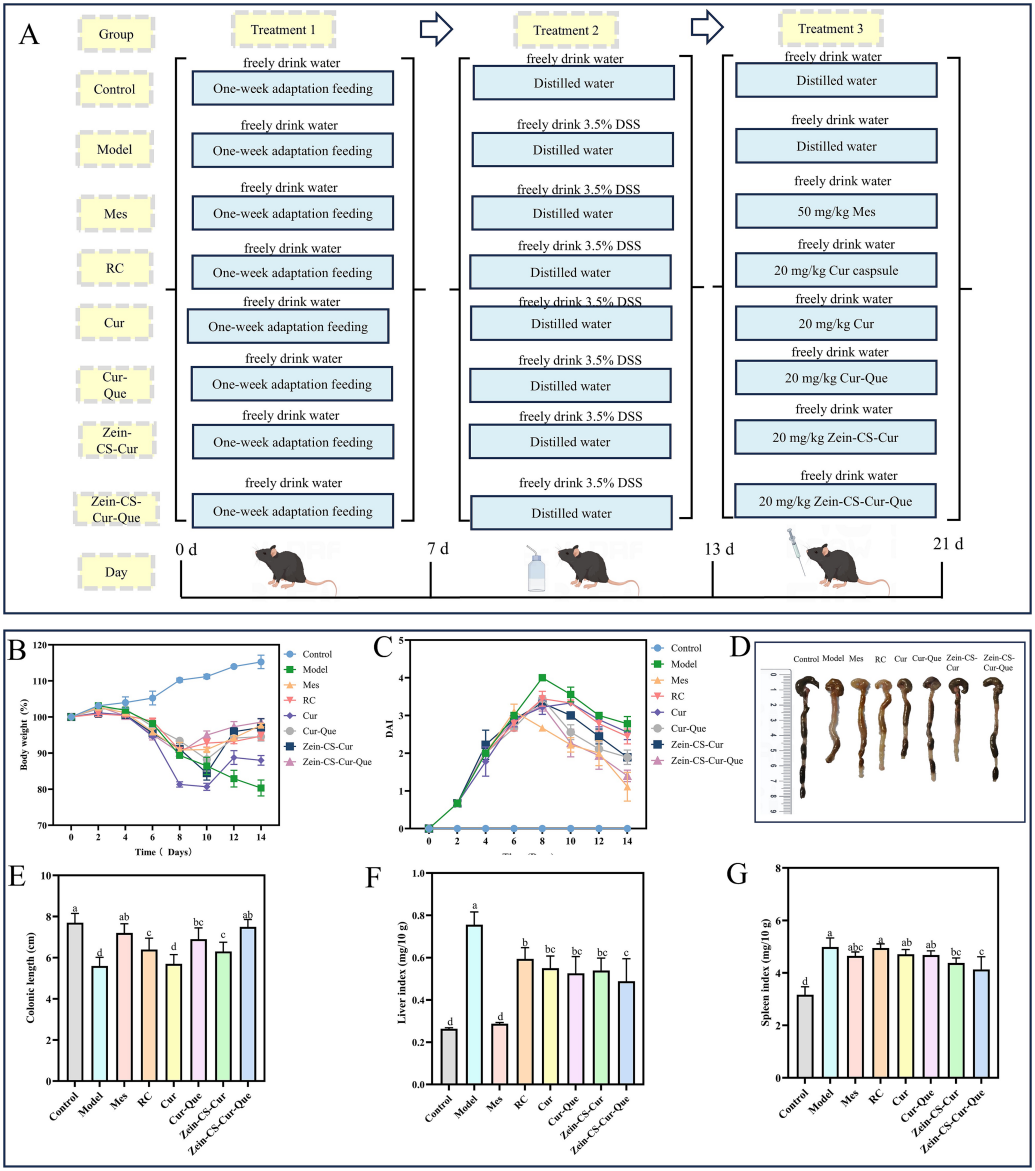
*In vivo* therapeutic effect of Zein-CS-Cur and Zein-CS-Cur-Que against UC. **(A)** Schematic diagram of DSS-induced ulcerative colitis modeling and grouping of different pharmacological interventions [(I) the normal control group (Control), (II) the DSS model group (Model), (III) the positive control group (Mes), (IV) the Israel curcumin capsule group (RC), (V) the curcumin group (Cur), (VI) the curcumin and quercetin group (Cur-Que), (VII) the curcumin-loaded nanoparticle group (Zein-CS-Cur), (VIII) the nanoparticles loaded with curcumin and quercetin (Zein-CS-Cur-Que). Drug gavage doses (Cur: 20 mg/kg/day)]. **(B)** Rate of change in body weight of mice during the modeling cycle, converted at 100% of the initial rate of change in body weight. **(C)** Disease activity index. **(D,E)** Schematic representation of the length of the resected colon in mice. **(F)** Liver index of mice, as liver mass over overall mass. **(G)** Spleen index of mice, as spleen mass over overall mass. (Different letters (a–d) indicate significant differences (*p* < 0.05) between groups).

All mice were maintained at a temperature of 25 ± 3 °C under a 12-h light/dark cycle. Mice were randomly divided into 8 groups with 10 mice in each group: (I) the normal control group (Control), (II) the DSS model group (Model), (III) the positive control group (Mes), (IV) the Israel curcumin capsule group (RC), (V) the curcumin group (Cur), (VI) the curcumin and quercetin group (Cur-Que), (VII) the curcumin-loaded nanoparticle group (Zein-CS-Cur), (VIII) the nanoparticles loaded with curcumin and quercetin (Zein-CS-Cur-Que). The flowchart illustrating the mice modeling and treatment process is presented in Figure 1A. Except for the Control group, the other groups were administered 3.5% DSS dissolved in 0.5% carboxymethylcellulose sodium for 6 days for modeling and then started administration to improve. The intervention groups (Mes, RC, Cur, Cur-Que, Zein-CS-Cur, Zein-CS-Cur-Que) were gavaged throughout the improvement cycle (7–14 days) at a dose of 200 μL. Mice in the positive group (Mes) received 50 mg/kg/day 5-aminosalicylic acid, and the other groups (RC, Cur, Cur-Que, Zein-CS-Cur, Zein-CS-Cur-Que) were given curcumin, curcumin-quercetin or curcumin-loaded nanoparticles with a gavage curcumin dose of 20 mg/kg/day and quercetin dose of 4 mg/kg/day. The blank control and model groups received an equivalent volume of sodium carboxymethylcellulose for the same duration.

Following modeling and drug improvement, the mice were subjected to a 24-h fasting period without water. The weight of mice was recorded every 3 days and stool samples were collected. After the experiment, collect the blood samples and calculate liver index, spleen index and colon index. The colon was photographed and measured, while a portion of the colon tissues underwent HE staining and immunohistochemical analysis using a 4% paraformaldehyde fixative.

### 2.4 Evaluation of the disease activity index

After acclimatization, the mice were observed and recorded daily for food and water intake, fecal occult blood, and body weight. Subsequently, feces were collected to assess occult blood status, and the rate of change in body weight and disease activity index (DAI) scores was calculated, where disease activity index (DAI) is defined as (Rate of loss of body weight and water intake score + Fecal trait score + Fecal occult blood score)/3.

### 2.5 Histopathology analysis

Colon tissues were collected, rinsed with saline, and immersed in 4% paraformaldehyde for 24 h for fixation. The tissues were then dehydrated, cleaned, infiltrated, paraffin-embedded, and stained with hematoxylin and eosin. Prepared samples were fixed onto slides and observed under a light microscope at 200× magnification. Images were captured, and the number of cup cells in an average of three crypts was counted.

### 2.6 Measurement of serum inflammatory cytokine and oxidative stress level

ELISA kits were used to determine levels of cytokines in serum of mice: tumor necrosis factor-α (TNF-α), interleukin-1β (IL-1β), interferon-γ (IFN-γ), interleukin-6 (IL-6) and interleukin-10 (IL-10). ELISA kits were used to determine oxidative stress level in colon tissue: myeloperoxidase (MPO), malondialdehyde (MDA), catalase (CAT) and superoxide dismutase (SOD).

### 2.7 Immunohistochemical analysis of colonic tissues

The colon tissue was embedded in paraffin, the specimen was dehydrated and sliced, and the antigen was repaired by water bath heating. After sealing, it was incubated with primary antibody at 4 °C overnight. Secondary antibodies were incubated, DAB color development was followed by hematoxylin re-staining, sealing, and subsequent observation under light microscopy.

### 2.8 16S rRNA sequencing of gut microbiota

According to the operating instructions of DNA extraction kit, the DNA of fecal flora was extracted. DNA was purified by gel electrophoresis, and PCR products were recovered by DNA gel recovery kit. The purified product was obtained through PCR amplification of the bacterial 16S rRNA V3–V4 region, using the genomic DNA as a template. Subsequently, data libraries were constructed, followed by high-throughput sequencing. The bacterial taxonomy and diversity were examined using the OTU table.

### 2.9 Real-time quantitative reverse transcription PCR

Total RNA was extracted from colon tissues utilizing the RNA Easy Fast Animal Tissue Extraction Kit, and its concentration and purity were evaluated. Using β-actin as the internal reference, cDNA was subjected to qPCR amplification. The relative expression levels of target genes in each sample were calculated using the 2^−ΔΔCT^ method. The primer sequences are presented in Table 1.

**Table 1:** Primers used for real-time PCR.

Genes	Primers	Primer sequences (5′–3′)
β-actin	F	5′-GGAGATTACTGCCCTGGCTCCTA
	R	5′-GACTCATCGTACTCCTGCTTGCTG
TLR4	F	5′-CTCACAACTTCAGTGGCTGGATTTA
	R	5′-GTCTCCACAGCCACCAGATTCTC
TRIF	F	5′-GCCAGCAACTTGGAAATCAGC
	R	5′-GGGGTCGTCACAGAGCTTG
NF-κB P65	F	5′-CATGCGTTTCCGTTACAAGTG
	R	5′-GTGCGTCTTAGTGGTATCTGTGCT
IκBα	F	5′-TTGGTGACTTTGGGTGCTGATG
	R	5′-CACACTTCAACAGGAGCGAGAC
MyD88	F	5′-CGACGCCTTCATCTGCTACTG
	R	5′-GCCGATAGTCTGTCTGTTCTAGTTG
STAT3	F	5′-ATGTCCTCTATCAGCACAACC
	R	5′-GACTCTTCCCACAGGCATCGG
SOCS1	F	5′-CGAGAACCTGGCACGCATCCCT
	R	5′-TCCACACCCACTCCCTCCGACCT
IL-6	F	5′-CTGCAAGAGACTTCCATCCAG
	R	5′-AGTGGTATAGACAGGTCTGTTGG

### 2.10 Short-chain fatty acids

Dissolve 100 mg of mouse feces in 450 μL methanol and 50 μL internal standard, stand at −20 °C for 60 min, and then centrifuge at 13,000 r/min for 15 min. After centrifugation, 25 mg anhydrous sodium sulfate was added and centrifuged again, and 100 μL supernatant was sucked to determine the content of short-chain fatty acids by gas chromatography-mass spectrometry.

### 2.11 Statistical analysis

The analysis of immunofluorescent proteins was performed to quantify the area of positive expression with ImageJ software. Experimental data were statistically analyzed using SPSS software, while correlation images were generated using GraphPad Prism version 9.0. Results are expressed as mean ± standard deviation (SD). Significant differences are indicated by the following markers: ^*^p < 0.05, ^**^p < 0.01, ^***^p < 0.001, and ^****^p < 0.0001. Different letters (a–h) indicate significant differences between groups.

## 3 Results and discussion

### 3.1 Therapeutic efficacy *in vivo*

The flow chart of the mouse modeling and treatment process is shown in Figure 1A. In order to investigate the effect of different treatment groups on the overall improvement of UC, several indexes such as the rate of change in body weight, DAI score, colon length, liver index, and spleen index were comprehensively evaluated (Figures 1B–G). Relative to Control, the model group showed significant weight loss, severe diarrhea, and visible bleeding in the feces, resulting in the highest DAI score in the model group during the test cycle. The Mes and Zein-CS-Cur-Que groups started to regain their weight on day 8 after gavage, whereas the Cur, Cur-Que, and Zein-CS-Cur groups did not start to regain their weight until day 10. And the decrease in DAI score showed the same trend after the drug intervention, indicating that the drug intervention group reduced the symptoms of colitis to some extent. The Zein-CS-Cur-Que group (*p* < 0.001) showed the same excellent improvement as the Mes group, which may be attributed to the targeted and slow release of curcumin and its high bioavailability, which slowed down the over digestion of curcumin by gastric acid to a certain extent.

This was also confirmed in the assessment of colon length, spleen index and liver index. In the model group, the colon was red, swollen and congested, the length was significantly shortened, and the liver and spleen appeared to be swollen, whereas Zein-CS-Cur-Que showed relatively excellent special effects in restoring the length of the colon and avoiding excessive swelling of the liver and spleen, which resulted in the least difference in the morphologic characteristics of the mice from the normal group of mice. Therefore, oral administration of Zein-CS-Cur-Que can effectively alleviate ulcerative colitis and restore intestinal damage.

### 3.2 Zein-CS-Cur-Que regulated intestinal inflammatory response

**Figure 2:**
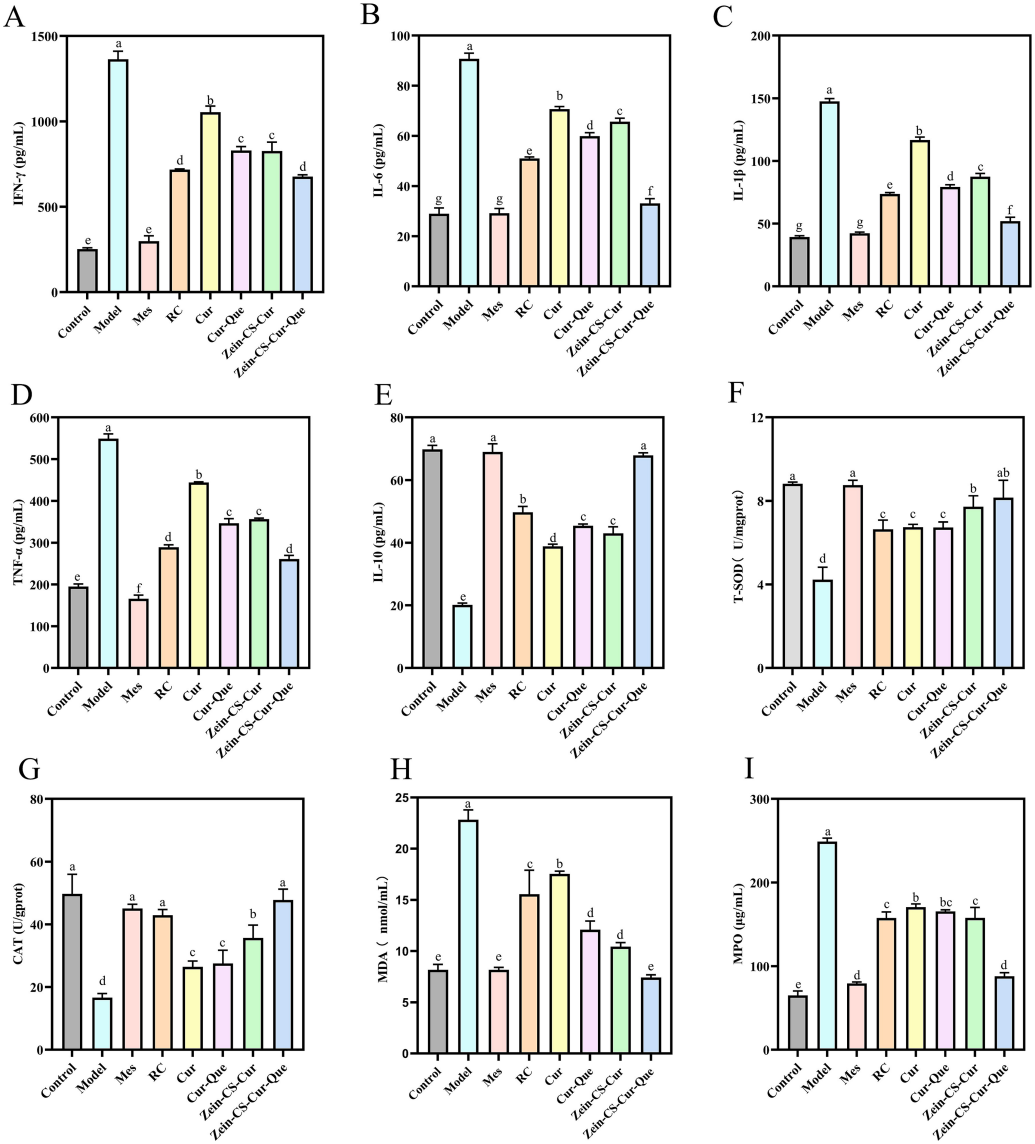
Assessment of the anti-inflammatory effect of RC, Cur, Cur-Que, Zein-CS-Cur and Zein-CS-Cur-Que on DSS-induced ulcerative colitis and oxidative stress *in vivo*. **(A)** INF-γ. **(B)** IL-6. **(C)** IL-1β. **(D)** TNF-α. **(E)** IL-10. **(F)** T-SOD. **(G)** CAT. **(H)** MDA. **(I)** MPO. Control is healthy mice, Model is 3.5% DSS-induced UC, and positive is Mes treatment. (Different letters (a–g) indicate significant differences (*p* < 0.05) between groups).

Inflammatory cytokines are closely related to intestinal macrophages, which can directly produce inflammatory injury to the intestinal tract and are strongly associated with the severity of UC (Figures 2A–E). Under normal circumstances, pro-inflammatory factors and anti-inflammatory factors in intestinal cavity are in a relatively balanced state, but ulcerative colitis can induce cell hypertrophy and activate T cells, which leads to the relative reduction of anti-inflammatory factors, and then cause colon tissue damage. Compared with the Control group, the levels of pro-inflammatory factors (IFN-γ, IL-6, IL-1β, and TNF-α), which promote intestinal mucosal injury and metaplastic necrosis, were significantly elevated in the Model. While the levels of anti-inflammatory factors (IL-10), which inhibit immune disorders, were significantly reduced. The plasma levels of inflammatory factors IFN-γ, IL-6, IL-1β, and TNF-α in Model group were 4.41, 2.21, 2.77 and 1.82 times higher than those in Control mice, respectively, the level of anti-inflammatory factor IL-10 decreased by 71.15% compared with the control mice (*p* < 0.001). Notably, compared with the Cur group, the RC, Cur-Que, Zein-CS-Cur and Zein-CS-Cur-Que groups showed better improvement, while the Zein-Cur-Que group was more able to regulate inflammatory cytokines back to normal levels, reduce inflammatory responses and maintain intestinal homeostasis.

Compared with the Cur group, the levels of IL-10 in the other intervention groups of RC, Cur-Que, Zein-CS-Cur and Zein-CS-Cur-Que increased by 28.94, 16.82, 10.64 and 74.56% (*p* < 0.001). These findings suggest that synergistic treatment or encapsulation enhances curcumin transportation *in vivo*, thereby reducing inflammation levels and restoring physiological functions.

### 3.3 Zein-CS-Cur-Que regulated intestinal oxidative stress

The results indicated a significant decrease (*p* < 0.001) in the activities of superoxide dismutase (SOD) and catalase (CAT), alongside a notable increase in malondialdehyde (MDA) and MPO levels within the model group (Figures 2F–I). This suggests that DSS exacerbates oxidative stress in mice, which subsequently contributes to the progression of intestinal inflammation. Upon measuring SOD and CAT levels, it was observed that the group treated solely with curcumin, as well as the Cur-Que group, exhibited comparatively lower oxidative stress levels than the model group. In contrast, the positive control group, along with the RC, Zein-Cur, and Zein-Cur-Que groups, demonstrated relatively elevated oxidative stress levels. These findings imply that the co-delivery of Zein-embedded curcumin and quercetin can effectively mitigate DSS induced oxidative stress.

### 3.4 Histologic analysis *in vivo*

**Figure 3:**
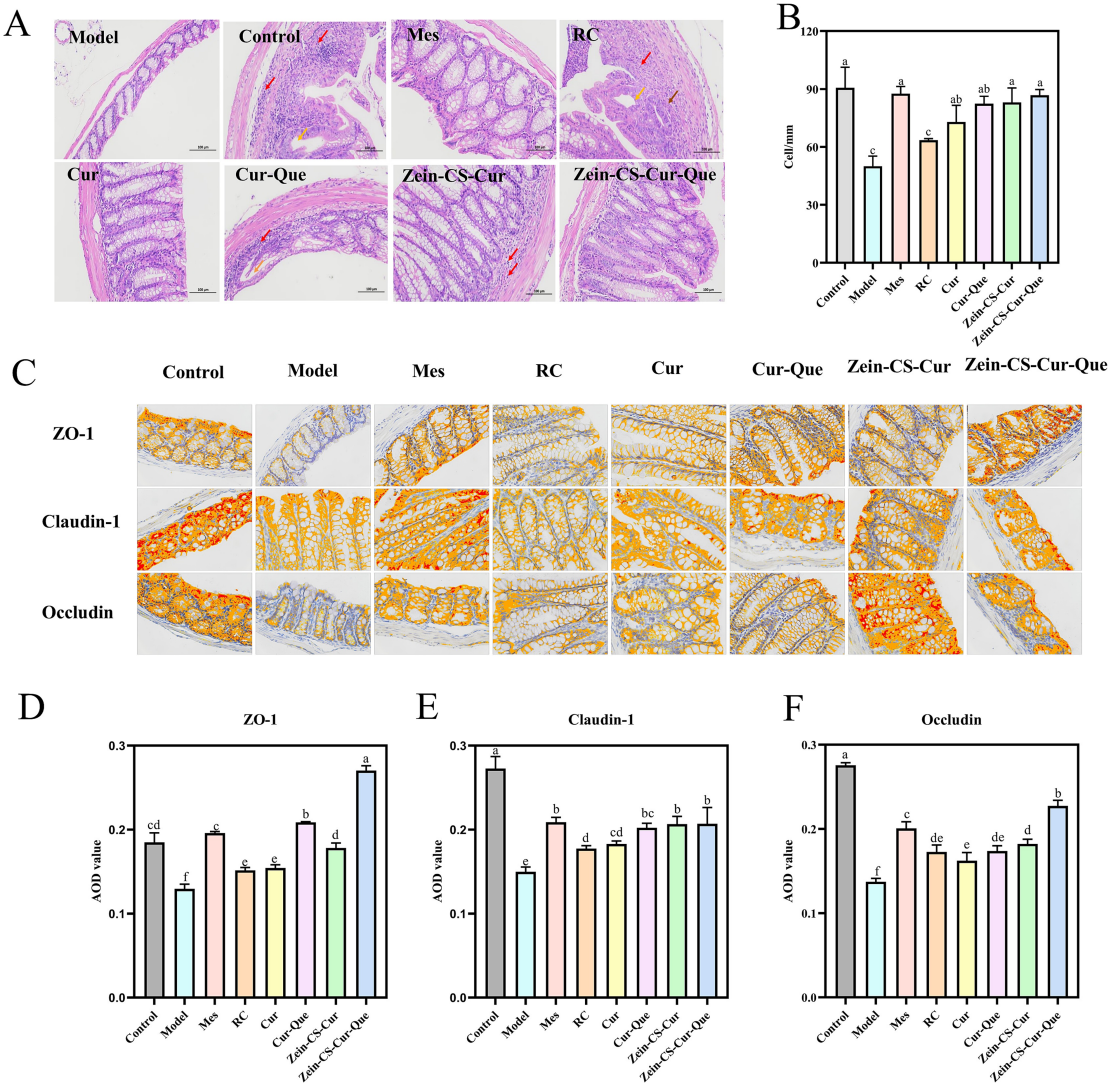
Repairing the intestinal barrier and maintaining intestinal homeostasis of RC, Cur, Cur-Que, Zein-CS-Cur and Zein-CS-Cur-Que. **(A)** H&E staining of the colon tissues and histological. Red arrows indicate inflammatory cell infiltration, brown arrows indicate tissue damage, and orange arrows indicate dilated intestinal glands. **(B)** Number of cup cells in colon tissue. **(C)** Representative images of ZO-1, Claudin-1, and Occludin protein expression in colon tissue. **(D)** Average optical density of ZO-1 (400×). **(E)** Average optical density of Claudin-1 (400×). **(F)** Average optical density of Occludin (400×). Control is healthy mice, Model is 3.5% DSS-induced UC, and positive is Mes treatment. (Different letters (a–f) indicate significant differences (*p* < 0.05) between groups).

In the Control group, a significant number of intestinal glands were observed in the lamina propria (Figure 3A), characterized by a dense arrangement, abundant cup cells, and loose connective tissue in the submucosal layer, with a clearly defined muscularis propria (25). Conversely, the Model group exhibited extensive ulcers, destruction of the intestinal gland structure, and mucosal epithelium, alongside infiltration of lymphocytes and granulocytes in both the lamina propria and submucosal layer (indicated by red arrows). In the RC and Cur groups, focal ulcers were noted in the intestinal tissue, accompanied by destruction of the intestinal gland structure and mucosal epithelium, which was replaced by a modest proliferation of fibrous connective tissue (brown arrows). Additionally, in these groups, the injury extended into the submucosa, with a minor infiltration of lymphocytes and granulocytes in the lamina propria and submucosa (red arrowheads). The Mes group, Cur-Que group, Zein-CS-Cur group, and Zein-CS-Cur-Que group displayed a substantial number of intestinal glands in the lamina propria, arranged densely, featuring an abundance of cup-shaped cells, along with occasional necrotic cell fragments. The submucosal layer consisted of sparse connective tissue, while the structure of the muscularis propria remained clear.

Colonic mucus depletion and a dramatic decrease in goblet cell density led to intestinal dysfunction and induce UC (26). Compared to the blank control group, the density of goblet cell in the model group of mice was significantly reduced (Figure 3B). In contrast, there was no significant difference in goblet cell density between the model group and the RC group. However, the goblet cell density in the Mes group, Cur group, Cur-Que group, Zein-CS-Cur group, and Zein-CS-Cur-Que group was significantly increased (*p* < 0.001).

### 3.5 Immunohistochemistry

Tight junction proteins, including ZO-1, Claudin-1, and Occludin, are critical components in the inflammatory cascade response associated with the pathogenesis of inflammatory bowel disease (IBD) (Figures 3C–F). The intestinal epithelial mechanical barrier plays a vital role in resisting inflammation, and the disruption of tight junction proteins is a significant contributor to intestinal barrier dysfunction. Immunohistochemical analysis showed that the expression of ZO-1, Occludin and Claudin-1 was significantly lower and disorganized, and the crypt structure was unclear in the Model group compared with the Control group. Furthermore, curcumin and quercetin have demonstrated the ability to reverse the reduction in the expression levels of tight junction proteins ZO-1 and Claudin-1. Notably, protein levels significantly increased following the synergistic action of both compounds or after the encapsulation of curcumin. The Zein-CS-Cur-Que effectively mitigated DSS-induced intestinal damage, with its protective effect ranking just below that of the Mes group.

### 3.6 16S rRNA sequencing of gut microbiota

**Figure 4:**
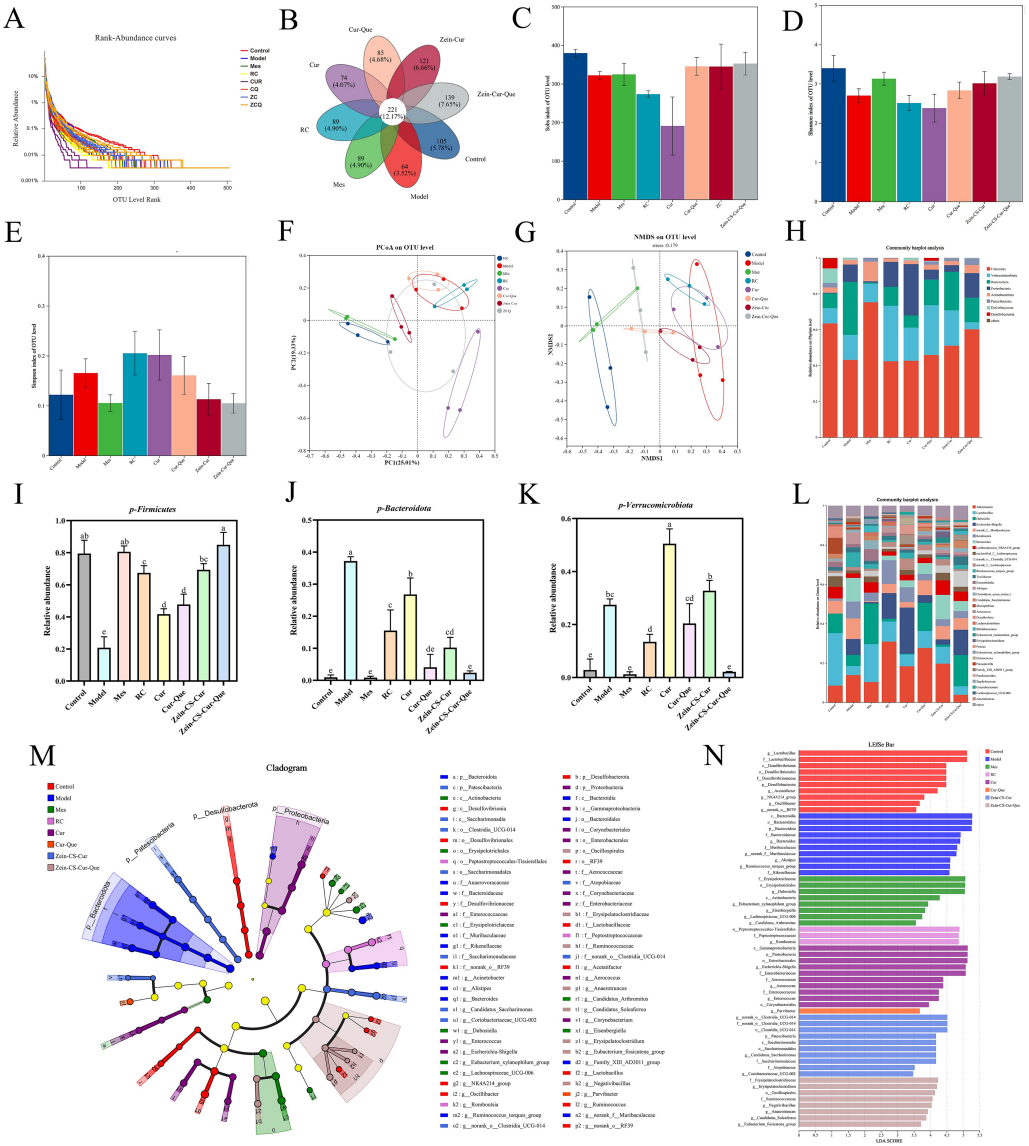
Evaluation of the effect of RC, Cur, Cur-Que, Zein-CS-Cur and Zein-CS-Cur-Que on intestinal flora and restoration of intestinal viability. **(A)** Dilution curve. **(B)** Venn diagram. **(C)** Sobs index. **(D)** Shannon index. **(E)** Simpson index. **(F)** PCOA. **(G)** NMDS. **(H)**. Histogram of microbial abundance at the phylum level. **(I)** Relative abundance of Firmicutes. **(J)** Relative abundance of Bacteroidetes. **(K)** Relative abundance of Verrucomicrobia. **(L)** Genus of microbial abundance at the phylum level. **(M)** LEfSe species difference analysis among the different formulations. **(N)** Linear discriminant analysis (LDA) results. (Different lowercase letters represent significant differences). Control is healthy mice, Model is 3.5% DSS-induced UC, and positive group is Mes treatment. (Different letters (a–f) indicate significant differences (*p* < 0.05) between groups).

UC induces disorders in intestinal flora and compromises intestinal barriers (27). To elucidate whether orally administered nanoparticles alter the intestinal flora in mice with colitis, we examined mouse feces using 16S rRNA high-throughput sequencing. The number of operational taxonomic units (OTUs) exhibited a significant upward trend with the gradual increase in sequencing depth, eventually reaching a plateau, indicating that the sequencing depth was adequate (Figure 4A). Venn analysis revealed that 5.78% of OTUs were identified in the Control, Model, Mes, RC, Cur, Cur-Que, Zein-CS-Cur, and Zein-CS-Cur-Que groups, respectively, with varying percentages of different OTUs: 3.52, 4.90, 4.90, 4.07, 4.68, 6.66 and 7.65% (Figure 4B). Compared to the blank group, the model group exhibited a significant decrease in the Shannon index and Sobs index (Figures 4C,D). Notably, the Zein-CS-Cur-Que significantly enhanced the correlation index compared to the curcumin group, demonstrating an opposing trend in Simpson’s index (Figure 4E). These findings suggest that the co-delivery of encapsulated pellets improves the structure of DSS-disturbed intestinal flora in mice, increasing species abundance and homogeneity within the sample communities.

To assess differences in species complexity between samples, beta diversity analysis was employed. Both principal coordinates analysis (PCOA) and non-metric multidimensional scaling (NMDS) were utilized to visualize the multivariate structure of the data and the variations within the gut microbiota (Figures 4F,G). A clear separation was observed between the blank and model groups, indicating significant differences in the overall composition of the gut microbiota. Notably, the Zein-CS-Cur-Que group showing a trend more closely resembling that of the healthy control group.

The imbalance of intestinal flora and the integrity of intestinal barriers can significantly influence the inflammatory response, contributing to the development of UC (28, 29). At the portal level, the predominant phyla include the thick-walled Firmicutes, Bacteroidota, Proteobacteria, and Verrucomicrobiota. However, the ecological imbalance observed in UC is characterized by a decrease in Firmicutes and an increase in Proteobacteria and Verrucomicrobiota, with these differences being statistically significant (30). Compared to the blank control group, the DSS induced colitis model exhibited a marked reduction in the abundance of Firmicutes, alongside a significant increase in the levels of Bacteroidota and Verrucomicrobiota (Figures 4H–K).

Analyzing the species composition at the genus taxonomic level, the analysis of variance at this level (Figure 4L) revealed a decrease in the relative abundance of beneficial bacteria such as Lactobacillus and Lachnospiraceae__NK4A136 in the model group compared to the normal group. Conversely, the relative abundance of Akkermansia, norank_f__Muribaculaceae, and Bacteroides increased (31, 32). Additionally, Zein-CS-Cur-Que was found to maintain the balance of intestinal flora, support intestinal homeostasis, and enhance the proportion of beneficial bacteria, thereby alleviating UC.

To determine the significant differences in species between groups, the results of the Linear discriminant analysis Effect Size indicated that a total of 63 taxa were identified, ranging from phylum to genus. The primary composition of the gut microbiota across the eight groups of mice consisted of Bacteroidota, Proteobacteria, and Verrucomicrobiota (Figure 4M). The linear discriminant analysis (LDA) revealed that the characteristic bacteria in the blank group were Desulfobacterota and norank_o__RF39, whereas Bacteroidota and the Ruminococcus_torques_group predominated in the model group (33, 34). Furthermore, the population of Erysipelotrichaceae significantly increased in the Mes group (Figure 4N), while Zein-CS-Cur-Que enhanced the abundance of beneficial bacteria, thereby alleviating UC.

### 3.7 Real-time quantitative reverse transcription PCR

**Figure 5:**
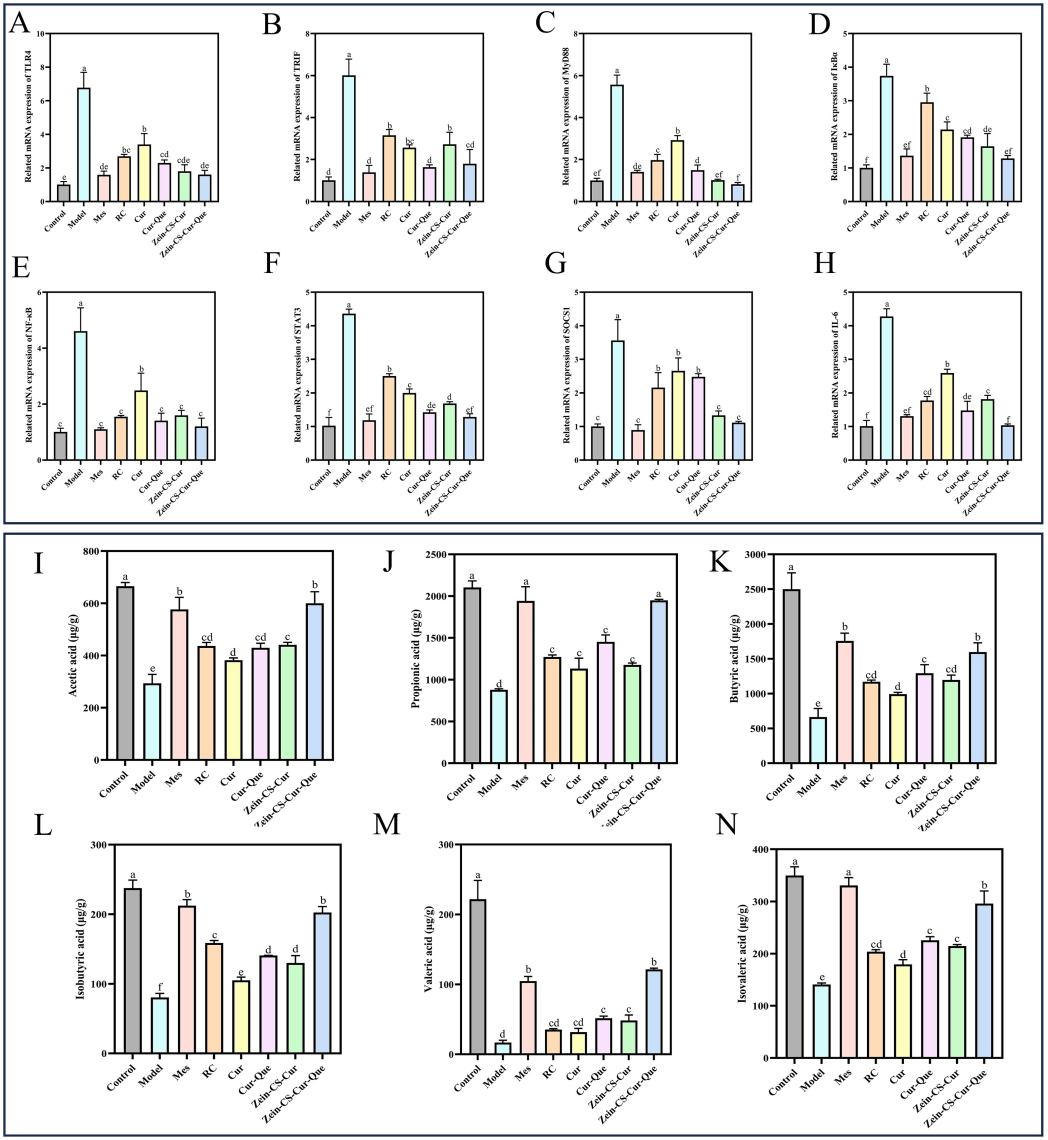
Zein-CS-Cur-Que alleviated colitis symptoms by inhibiting the TLR4/NF-κB/JAK2/STAT3 pathways and promoting the production of short-chain fatty acids. **(A)** TLR4. **(B)** TRIF. **(C)** MyD88. **(D)** IκBα. **(E)** NF-κB p65. **(F)** STAT3. **(G)** SOCS1. **(H)** IL-6. **(I)** Acetic acid. **(J)** Propionic acid. **(K)** Butyric acid. **(L)** Isobutyric acid. **(M)** Valeric acid. **(N)** Isovaleric acid. Control is healthy mice, Model is 3.5% DSS-induced UC, and positive is Mes treatment. (Different letters (a–g) indicate significant differences (*p* < 0.05) between groups).

Numerous studies have shown that the TLR4/NF-κB signaling pathway is an important mechanism to increase the expression of pro-inflammatory factors, and that molecular interactions between JAK2 and STAT3 regulate the transcription of target genes, these two signaling pathways play an important role in the pathological mechanisms of UC, and together they maintain intestinal homeostasis (35, 36). The expression levels of TLR4, TRIF, MyD88, IκBα, and NF-κB p65 in the TLR4/MyD88/NF-κB pathway (Figures 5A–E), as well as STAT3, SOCS1, and IL-6 in the JAK2/STAT3 pathway in colonic tissue (Figures 5F–H), were assessed using RT-qPCR technology. These findings suggest a robust association between these two pathways in ameliorating UC. The mRNA expression levels in the Model group and various dosage improvement groups were significantly elevated compared to the normal control group, indicating a substantial increase in inflammatory-related protein genes. Notably, the relative expression levels of protein genes associated with Cur-Que, Zein-CS-Cur, and Zein-CS-Cur-Que were lower than in the Cur and RC groups, and ultimately the nanoparticles had the best overall effect on the improvement of ulcerative colitis, which is in line with immunohistochemistry results analyzed previously.

### 3.8 Short-chain fatty acid

Dietary fiber, shed epithelial cells, lysed bacteria and other substances in the intestine can be fermented by intestinal bacteria to produce short-chain fatty acids, including acetic acid, propionic acid, isobutyric acid, butyric acid, isovaleric acid, valeric acid and hexanoic acid, which are metabolites of intestinal flora that maintain intestinal homeostasis and exert anti-inflammatory effects (37, 38). Significantly lower levels of acetic, propionic, n-butyric, isobutyric, n-pentanoic, and isovaleric acids in Model compared to Control (Figures 5I–N). Compared with the Cur group, the content of SCFAs in the Cur-Que, Zein-CS-Cur and Zein-CS-Cur-Que groups was significantly increased, which indicated that Cur was able to initially anti-inflammatory and maintain intestinal impermeability, and that after synergistic treatment with Cur and Que. or synergistic embedding, it was able to be beneficial to the survival of intestinal microorganisms, maintain the vitality of intestinal immune cells, and alleviate intestinal damage caused by UC (39, 40).

## 4 Conclusion

In summary, we successfully synthesized new nanoparticles composed of Zein-CS-Cur-Que to enhance the hydrophobicity and anti-inflammatory properties of curcumin and quercetin. The UC mouse model was established, and the therapeutic effects of various drugs were evaluated. It was successfully confirmed that the established Zein-CS-Cur-Que could relieve ulcerative colitis in many ways. The specific performance and outstanding contributions are as follows: Zein-CS-Cur-Que can regulate inflammatory factors (reducing the expression of pro-inflammatory factors such as IL-6, TNF-α, and IL-1β), balance the level of oxidative stress, repair the intestinal barrier (up-regulating the expression levels of ZO-1, Claudin-1, and Occludin), increase the intestinal flora (Firmicutes and Bacteroidetes), and promote short-chain fatty acid production through nutritional intervention. The nanocomposite system developed in this study is thus crucial for the development of functional foods, dietary supplements and the advancement of precision nanomedicine for ulcerative colitis. Novel insights from this study provide a promising new nutrition intervention strategy for treatment of UC based on co-delivery of food bioactive using Zein-CS-Cur-Que.

## Data Availability

The original contributions presented in the study are included in the article/supplementary material, further inquiries can be directed to the corresponding authors.
